# Synthesis of Starfish-Shaped ZnS Nanostructures by Hydrothermal Method and Their Electrochemical Sensing of Dopamine

**DOI:** 10.7759/cureus.70481

**Published:** 2024-09-30

**Authors:** Deepika Dinesh, Sherin Celshia, Muthamizh Selvamani, Vasugi Suresh, Mohammed Asif Hussein

**Affiliations:** 1 Physiology, Saveetha Dental College and Hospitals, Saveetha Institute of Medical and Technical Sciences, Saveetha University, Chennai, IND

**Keywords:** dopamine, hydrothermal, nanostructure, starfish-shaped, zns

## Abstract

Introduction

Dopamine serves an essential function as a neurotransmitter, influencing the regulation of movement, cognitive processes, and emotional states. The identification of abnormal dopamine levels is critical for clinical diagnoses and scientific research, given its links to various disorders, including depression, schizophrenia, and Parkinson's disease. The distinctive electrochemical characteristics, stability, and broad bandgap of zinc sulfide (ZnS) nanostructures render them particularly fascinating. The hydrothermal method is recognized as an effective and economical approach for the fabrication of ZnS nanostructures, exhibiting a range of morphologies. Utilizing this method to create ZnS nanostructures leads to the formation of structures characterized by extensive surface areas, hierarchical designs, and improved electrochemical properties.

Aim

The objective is to examine the electrochemical characteristics of ZnS starfish-shaped nanostructures produced through the hydrothermal technique and to assess their viability as a sensing platform for dopamine detection.

Materials and methods

To synthesize ZnS nanoflowers, stoichiometric amounts of transition metal salts were prepared: 10 mM of Zn(NO_3_)_2_•3H_2_O and 30 mM of sodium thiosulfate (Na_2_S_2_O_3_•5H_2_O) were dissolved in 30 mL of deionized water and stirred for 20 minutes. The solutions were then combined and transferred into a 100 mL Teflon autoclave reactor, which was heated at 200 °C for 12 hours in a furnace. This process utilized the hydrothermal technique to produce the desired ZnS nanoflowers.

Result

The crystalline arrangement of ZnS was validated by X-ray diffraction (XRD) analysis, aligning with the Joint Committee on Powder Diffraction Standards (JCPDS). Moreover, field emission scanning electron microscopy (FE-SEM) illustrated the particle morphology of ZnS, showing a range between 200 and 500 nm size. Additionally, the cyclic voltammetry results indicated that the modified electrode produced a greater current response than the bare electrode, highlighting its improved sensitivity to dopamine molecules.

Conclusion

ZnS nanoparticles were synthesized via a hydrothermal method and characterized using XRD and FE-SEM. These nanoparticles were used for electrochemical dopamine detection, showing potential for advanced sensing platforms. Integrating ZnS into microfluidic devices enables real-time dopamine monitoring, opening new possibilities for healthcare and neurochemical research. Exploring surface engineering techniques could further enhance the electrochemical performance of ZnS-based sensors.

## Introduction

In recent years, the development of nanomaterials has greatly improved the performance of electrochemical sensors, enabling swift and precise detection of specific analytes. These sensors have become valuable tools for identifying and quantifying pharmaceutical compounds, thanks to their ease of use, affordability, and exceptional sensitivity. Among the diverse materials investigated for sensing applications, metal oxides have attracted considerable interest due to their distinctive physicochemical characteristics and capabilities in electrochemical sensing [1].

Nanomaterials have attracted significant attention in various fields due to their unique properties and potential applications. Nanoparticles serve as an excellent choice for improving the performance of electrochemical sensors, thanks to their outstanding features, such as a high surface-area-to-volume ratio, the ability to be tailored in size and shape, and impressive catalytic capabilities [2]. Electrochemical sensing is a powerful analytical technique that involves the application of electrochemical methods to detect and quantify the presence of chemical species in a sample. This sensing technique relies on the principle of converting a chemical signal into an electrical signal, making it widely used in various fields, including environmental monitoring, biomedical applications, food safety, and industrial process control [3]. Zinc sulfide (ZnS) nanostructures have gained considerable interest due to their wide bandgap, high stability, and excellent electrochemical properties. Working electrodes can have their surface modified using ZnS nanoparticles. This alteration increases the electrode's electrocatalytic activity, improving electron transport and increasing the sensitivity of target analyte detection [4].

Biosensors are devices designed to identify particular substances within a sample by employing electrochemical, optical, or various other transducers alongside a biological recognition system, which converts the concentration of the substance into an electrical signal. These instruments are extensively utilized in fields such as bioprocessing, medical diagnostics, agriculture, and environmental monitoring [5]. The hydrothermal method offers numerous advantages over alternative techniques. It enables the production of nanomaterials with elevated vapor pressures while minimizing material loss. Additionally, this method allows for the generation of typically unstable nanoparticles at elevated temperatures. Furthermore, it provides precise control over particle size, distribution, phase homogeneity, and morphology [6,7]. Hydrothermal synthesis is a method employed to create nanoparticles through chemical reactions taking place in a water-based solution at specific temperature and pressure settings [6]. This technology is commonly employed in the fabrication of a wide range of materials characterized by specific size, shape, and composition, such as metal oxides, metal sulfides, semiconductors, and nanomaterials.

In the field of nanoparticle synthesis, various forms have been recognized, including nanopowders, nanotubes, nanowires, nanoclusters, nanosheets, and nanorods. Although nanoflowers are not as widely acknowledged as nanowires or carbon nanotubes, which are more prevalent, they have attracted interest due to their current and prospective applications in optoelectronic devices, sensors, catalysis, and solar cells. ZnS is a chemical compound consisting of zinc and sulfur. It is an inorganic binary compound that occurs naturally as the mineral sphalerite. ZnS is classified as a quantum-confined nanocluster or quantum dot, which refers to tiny spheres (approximately 10-100 Å in diameter) of a conventional semiconductor. ZnS has a bandgap of 3.66 eV at 300 K, making it suitable for ultraviolet (UV) radiation in optical interband transitions [8]. ZnS can exist in two distinct crystal forms: zinc blende and wurtzite. Both structures exhibit an identical bandgap energy of 3.68 eV and possess a direct band structure [9]. The technology is widely employed to manufacture a diverse array of materials characterized by specific dimensions, shapes, and compositions, including metal oxides, metal sulfides, semiconductors, and nanomaterials.

Dopamine, a type of neurotransmitter, is categorized as both excitatory and inhibitory in nature. It is essential for the regulation of attention, cognitive functions, pleasure, motor activity, and hormonal processes. This important hormone is vital for the management of neuronal activity and metabolic functions. Derived from the amino acid tyrosine, dopamine is a catecholamine that is synthesized in the substantia nigra and the ventral tegmental area [2,8,11]. Electrochemical sensing is a powerful analytical method that leverages electrochemical characteristics to identify and measure various analytes. The conversion of a chemical signal to an electrical signal occurs at the interface between the electrode and electrolyte. Typically, an electrochemical sensor comprises three essential components: a working electrode, a reference electrode, and a counter electrode [12]. The advancement and diversification of potential applications for functional nanomaterials necessitate the creation of user-friendly, rapid, and eco-friendly synthetic methods. A closed system utilizing water as a solvent can facilitate the reaction under controlled temperature and pressure, thereby mimicking the natural mineralization process of crystal formation. The physical properties of water, including vapor pressure, density, surface tension, viscosity, and ionic product, are significantly influenced by hydrothermal conditions.

The hydrothermal method was first introduced in the crystallization process in 1882. This technique not only yields products with high crystallinity and a narrow size distribution but also ensures high purity and minimal aggregation while effectively lowering the reaction temperature of the systems involved [5]. Electrochemical sensors consist of two categories: biosensors and chemical sensors. Chemical sensors, unlike biosensors, consist of non-biologically active components, which enhance their sensitivity and selectivity in detecting analytes. Modified electrodes are commonly utilized as sensing components in electrochemical sensors. These modified electrodes can be created using various inorganic or organic materials known for their excellent electrical conductivity and catalytic properties. The present research endeavors to synthesize ZnS starfish-like nanostructures through the hydrothermal method and subsequently apply them for electrochemical detection of dopamine.

## Materials and methods

Synthesis of ZnS nanoparticles

**Figure 1:**
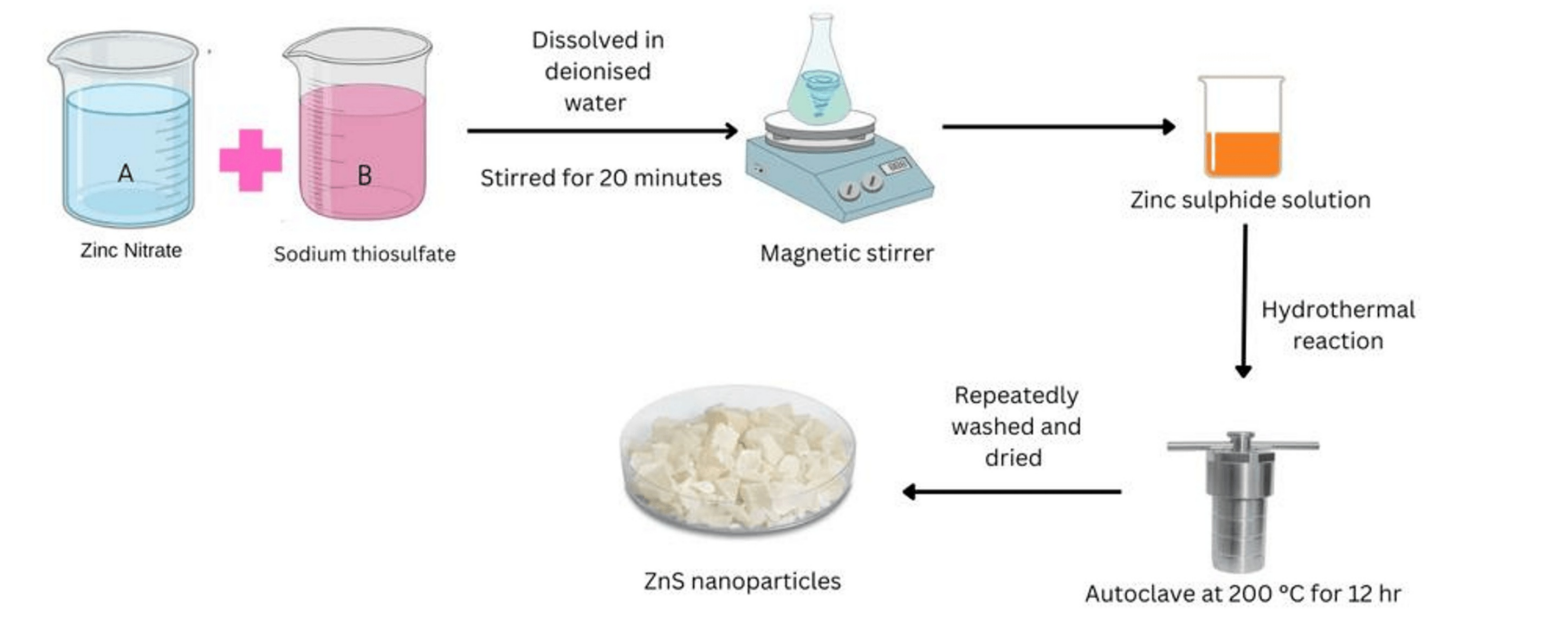
The process of synthesizing the required ZnS nanoparticles through the hydrothermal technique. ZnS: zinc sulfide

For the synthesis of ZnS nanoflowers, stoichiometric amounts of the transition metal salts 10 mM of Zn(NO_3_)2•3H_2_O and 30 mM of sodium thiosulfate (Na_2_S_2_O_3_•5H_2_O) were dissolved in 30 mL of deionized water and stirred for 20 minutes. The solutions were mixed and transferred into a 100 mL Teflon autoclave reactor and heated at 200 °C for 12 hours in a furnace. The process of synthesizing the required ZnS nanoflower through the hydrothermal technique is depicted in Figure 1.

Electrode preparation

Glassy Carbon Electrode (GCE)

The electrochemical detection process was modified with ZnS. Prior to this modification, the working electrode was mechanically polished with 1 µm, 0.3 µm, and 0.05 µm alumina pastes for mirror finishing. Subsequently, it was subjected to ultrasonication in double-distilled water for several minutes to clean the surface of the GCE. The ZnS suspension was prepared by dispersing 5 mg of ZnS in 10 mL of ethanol for 20 minutes of ultrasonic agitation. Subsequently, the GCE was coated with 10 µL of the suspension using the drop coating method and then air-dried. The fabricated working electrode was used to sense dopamine by electrochemical method.

## Results

XRD analysis

**Figure 2:**
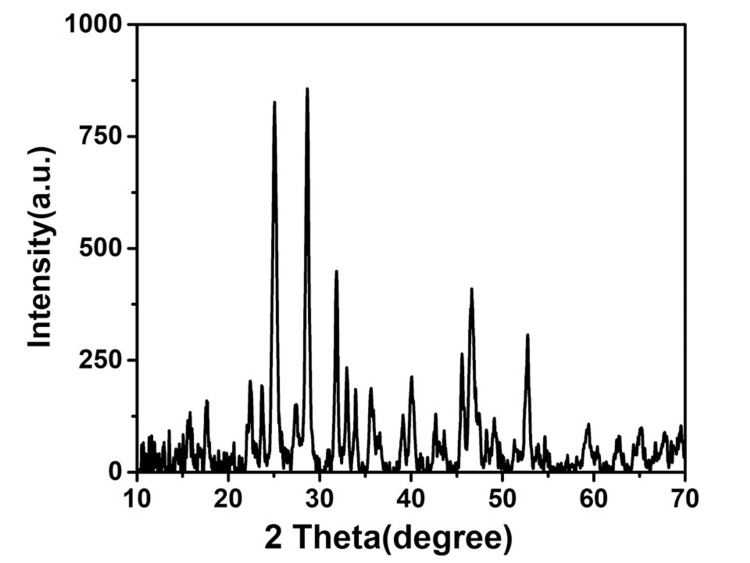
XRD pattern of the ZnS nanostructure. XRD: X-ray diffraction; ZnS: zinc sulfide

The detailed information about the crystallographic structure, chemical composition, and phase purity of the ZnS was investigated using XRD analysis, as shown in Figure 2. XRD works by irradiating a material with incident X-rays and then measuring the intensities and scattering angles of the X-rays that leave the material. XRD is based on constructive interference of monochromatic X-rays and a crystalline sample. These X-rays are generated by a cathode ray tube, filtered to produce monochromatic radiation, collimated to concentrate, and directed toward the sample. The XRD analysis of ZnS showed sharp peaks, and the pattern was confirmed with the reference pattern of ZnS (JCPDS, Joint Committee on Powder Diffraction Standards 36-1450) [13].

Morphological analysis

**Figure 3:**
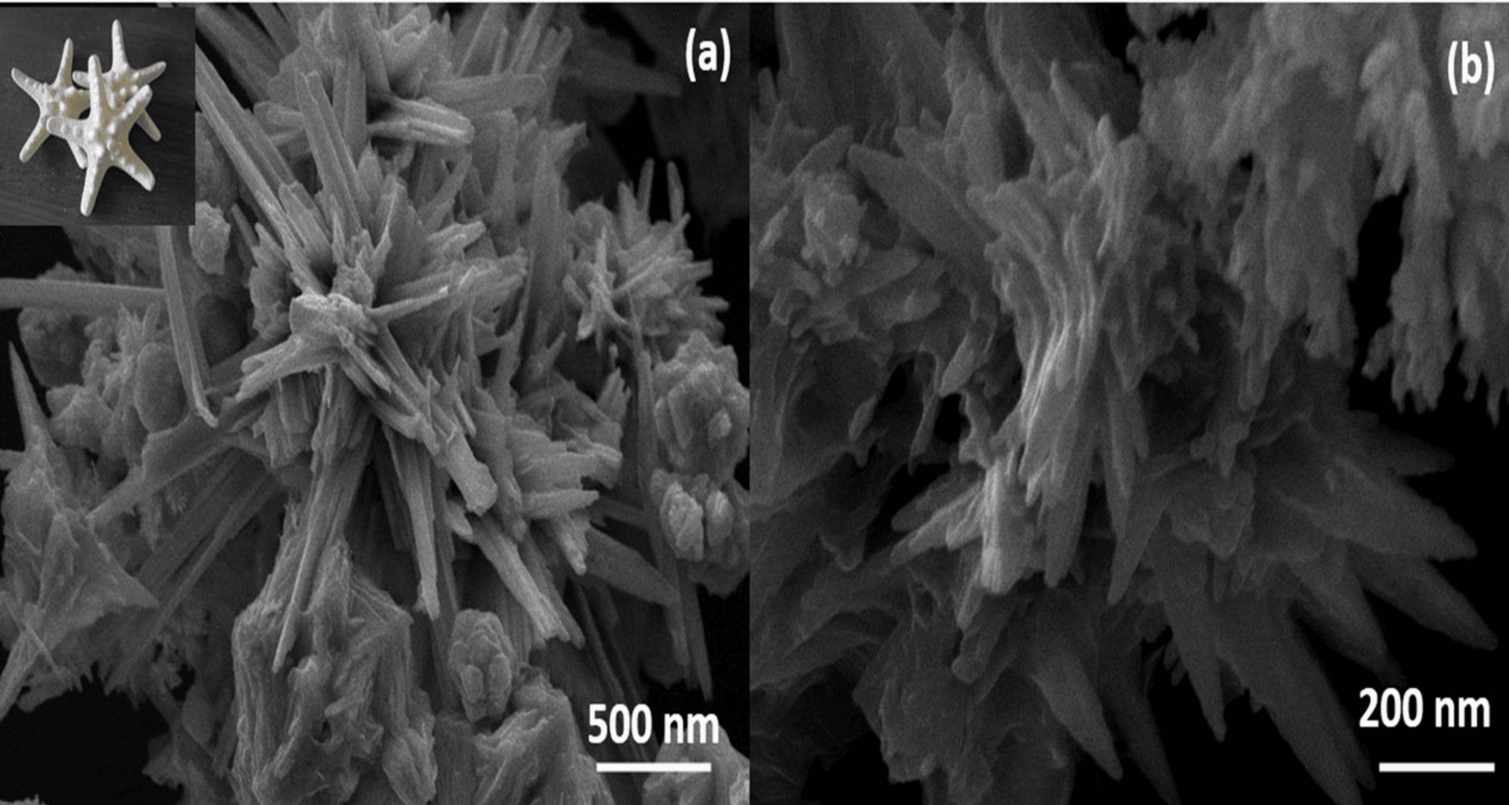
The FE-SEM images of the ZnS nanostructure prepared by hydrothermal treatment. (a) ZnS at 500 nm; (b) ZnS at 200 nm FE-SEM: field emission scanning electron microscopy; ZnS: zinc sulfide

Field emission scanning electron microscopy (FE-SEM) is an advanced technology used to capture the microstructure image of the materials. Figures 3a, 3b show the FE-SEM analysis. The starfish-like morphology of ZnS was observed at 500 nm and 200 nm, respectively.

Cyclic voltammetry

**Figure 4:**
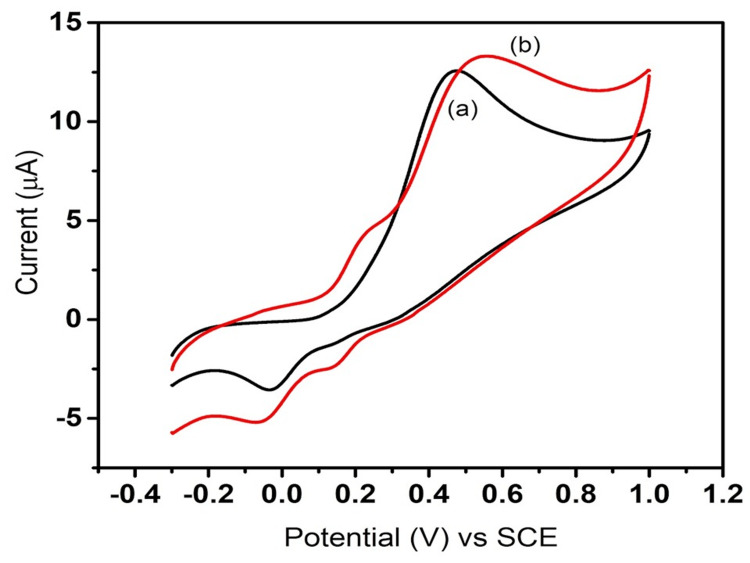
Cyclic voltammetry response of the bare electrode and ZnS-modified electrode towards dopamine. Line (a) represents the unmodified GCE sensing towards dopamine (black line), while (b) is the ZnS-modified GCE sensing towards dopamine (red line) ZnS: zinc sulfide; SCE: saturated calomel electrode

Cyclic voltammetry (CV) is an electrochemical technique that measures the current that develops in an electrochemical cell under conditions where voltage is applied. CV is performed by cycling the potential of a working electrode, and measuring the resulting current. Figure 4 shows the CV response of the modified electrode with ZnS at various potentials. In Figure 4, the black line (a) denotes the current response of the standard saturated calomel electrode (SCE) that is at the potential of 0.46 V; the current response is 12.60 μA. The red line (b) denotes the current response of the electrode modified with ZnS nanostructure suspension that is at the potential of 0.54 V, and the current response is 13.38 μA in response to an applied potential of 50 mV/second.

**Figure 5:**
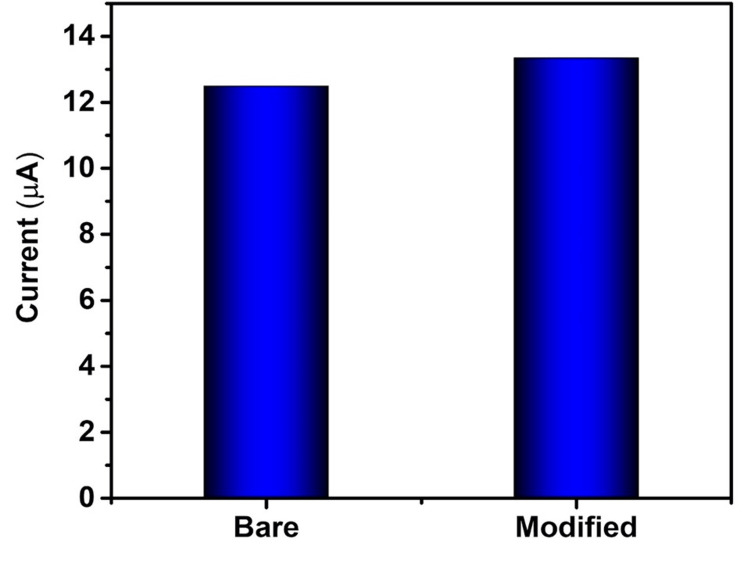
Cyclic voltammetry current response of the bare and ZnS-modified electrode towards dopamine. ZnS: zinc sulfide

For the bare electrode, a potential of 50 mV/second resulted in a corresponding current response of 12.60 μA, while for the electrode modified with ZnS, the application of a potential of 50 mV/second led to a corresponding current response of 13.38 μA (Figure 5). Compared to unmodified and modified GCE towards dopamine sensing, an increased current response was observed for the nanostructure-modified electrode than the unmodified electrode, indicating the influence of ZnS coating on the working electrode towards dopamine sensing by electrochemical method. The CV response shows that compared to the bare electrode, the modified electrode showed a higher current response, showing higher sensing of ZnS towards dopamine [14].

**Figure 6:**
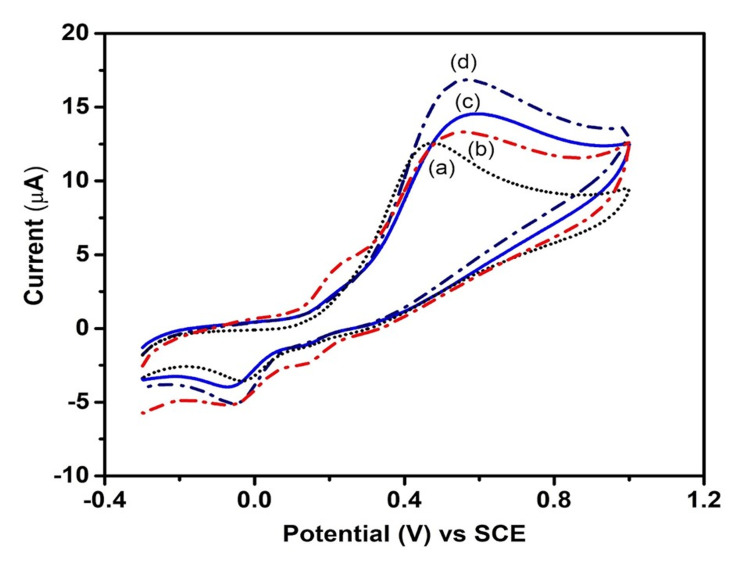
Cyclic voltammetry response of the bare electrode and the ZnS-modified electrode potentials towards dopamine. Applied potentials of (a) 50 mV/second, (b) 60 mV/second, (c) 70 mV/second, (d) applied potential of 80 mV/second ZnS: zinc sulfide; SCE: saturated calomel electrode

The CV response of the bare electrode and ZnS-modified electrode at the various applied potentials of 50-80 mV/second. At each applied potential, the ZnS-modified electrode shows a shift in potential as well as an increase in current response. At an applied potential of 50 mV/second, it shows a current response of 12.56 μA; at 60 mV/second, it shows a current response of 13.28 μA; at 70 mV/second, it shows a current response of 14.06 μA; and at 80 mV/second, it shows a current response of 17.01 μA. From the above observation, we can conclude that an increase in the applied potential results in the corresponding increase in the current response, which indicates the good sensing ability of the ZnS nanostructure towards dopamine sensing by electrochemical method (Figure 6).

## Discussion

This study focused on the synthesis of ZnS nanostructure through the hydrothermal method, followed by an assessment of their sensitivity to dopamine. ZnS is recognized as a semiconductor material that exhibits two primary phases: the cubic zinc blended phase and the hexagonal wurtzite phase, both of which possess a notable exciton binding energy. The ability to manipulate the size, shape, and crystalline structure of ZnS nanostructure has attracted considerable research interest due to the advantageous physical properties that result. These nanostructures serve various functions, including optical sensing, catalysis, and photoconductivity. The importance of inorganic nanomaterials has gained increasing recognition, particularly for their potential applications in photocatalysis, such as the degradation of organic pollutants and hydrogen production via water splitting. As a result, significant efforts have been made in recent decades to advance ZnS nanostructures, aiming to enhance their photocatalytic efficiency by increasing surface area and improving charge separation [15].

Elif et al. conducted a study focused on the detection of dopamine through the use of ZnS nanostructure affixed to a composite graphene paper electrode. The findings indicated that the composite graphene paper electrode exhibited markedly enhanced catalytic efficiency in the electrochemical oxidation of dopamine relative to the standard graphene paper electrode. The researchers determined that the composite graphene paper electrode could selectively detect dopamine, even in the presence of ascorbic acid and uric acid [16].

Figure 1 presents the XRD pattern of ZnS nanoparticles that were synthesized using a hydrothermal method. The composition and properties of the ZnS particles were analyzed through XRD, with the resulting peaks being compared to the standard JCPDS no. 80-0020, confirming a successful match. The prominent peaks observed indicate a high degree of crystallinity in the synthesized ZnS compound. Figure 2 showcases FE-SEM images of the ZnS particles. FE-SEM is an advanced technique employed to capture microstructural images of materials, typically performed in a high vacuum to avoid interference from gas molecules with the electron beam and to utilize emitted secondary and backscattered electrons for imaging purposes. The ZnS nanoparticles exhibit a unique flower-like morphology. ZnS can exist in two crystalline forms: wurtzite, which has a hexagonal structure, and zinc blende (sphalerite), which has a cubic structure. Figures 3, 4 illustrate the CV response of the synthesized ZnS nanoparticles. CV is an electrochemical technique that investigates the reduction and oxidation processes of molecular species by applying a potential waveform to an electrochemical cell and measuring the resulting current response.

In a separate study conducted by Hong et al., a three-dimensional graphene foam (3D GF) was created through chemical vapor deposition. ZnS nanostructures were grown in situ on the surface of the 3D GF (ZnS NPs/3D GF) using a straightforward hydrothermal method for the simultaneous detection of levodopa and uric acid. The results indicate that a porous mesh film composed of ZnS NPs GF electrodes exhibits high sensitivity in the detection of both levodopa and uric acid [17]. A separate study conducted by Reddy et al. focused on the application of CuO as a sensor for dopamine detection. In this research, rod-shaped CuO nanoparticles were synthesized via an annealing process in an air environment, utilizing copper plates as the starting material. Furthermore, flake-shaped CuO nanoparticles were created through a hydrothermal approach. The findings indicated that the modified carbon paste displayed significant sensing performance for the electrochemical analysis of dopamine in the presence of ascorbic acid. The CuO nanoparticle-modified carbon paste electrode exhibited a lower detection limit than those reported in earlier studies [18].

Limitations

The hydrothermal method approach used to produce starfish-shaped ZnS nanostructures may encounter issues with scalability, which could hinder the ability to generate consistent nanostructures in large volumes. Furthermore, ZnS nanostructures may experience stability problems when exposed to different environmental conditions, potentially compromising their durability and effectiveness over time. Another difficulty lies in achieving effective surface functionalization, which can restrict the nanostructures' sensitivity and selectivity for dopamine detection in electrochemical applications. Fluctuations in the hydrothermal method parameters may result in variations in the size and shape of the ZnS nanostructures, impacting the reproducibility of the results. Finally, the electrochemical detection of dopamine may be challenged by interference from other electroactive compounds, which can complicate the precise measurement and analysis of dopamine in complex samples.

## Conclusions

ZnS was synthesized through the hydrothermal method, with the formation of nanoparticles confirmed by XRD analysis and their morphology validated using FE-SEM. The resultant nanomaterial was subsequently employed for the electrochemical detection of dopamine. The production of ZnS offers a promising avenue for the development of enhanced dopamine-sensing platforms. Additionally, the incorporation of this nanoparticle into microfluidic devices facilitates the real-time monitoring of dopamine concentrations, thus paving the way for innovative applications in healthcare and neurochemical studies. Investigating various surface engineering techniques and their impact on the electrochemical characteristics of ZnS may lead to the advancement of more sophisticated dopamine sensors.
